# Supplementary material for: Precision engineering of nano-assemblies in superfluid helium by the use of van der Waals forces
Source: Commun Chem. 2024 Jun 4;7:125. doi: 10.1038/s42004-024-01203-5 (PMC11150505; doi:10.1038/s42004-024-01203-5)
Supplement: Supplementary file 2 — Supplementary Information [file 42004_2024_1203_MOESM2_ESM.pdf]

Precision engineering of nano-assemblies in superfluid helium by the use of weak van der Waals forces

*Gokhan Topcu,[a] Aula M. A. Al Hindawi,[a, b] Cheng Feng,[a] Daniel Spence,[a] Berlian Sitorus,[a, c] Hanqing Liu,[a] Andrew M. Ellis[a] and Shengfu Yang[a] **

[a] *School of Chemistry, University of Leicester, Leicester, LE1 7RH, UK.*

[b] *Department of Chemistry, College of Education for Pure Science, University of Karbala, Karbala, Iraq*

[c] *Department of Chemistry, Tanjungpura University, Pontianak, Indonesia.*

Supplementary Figures

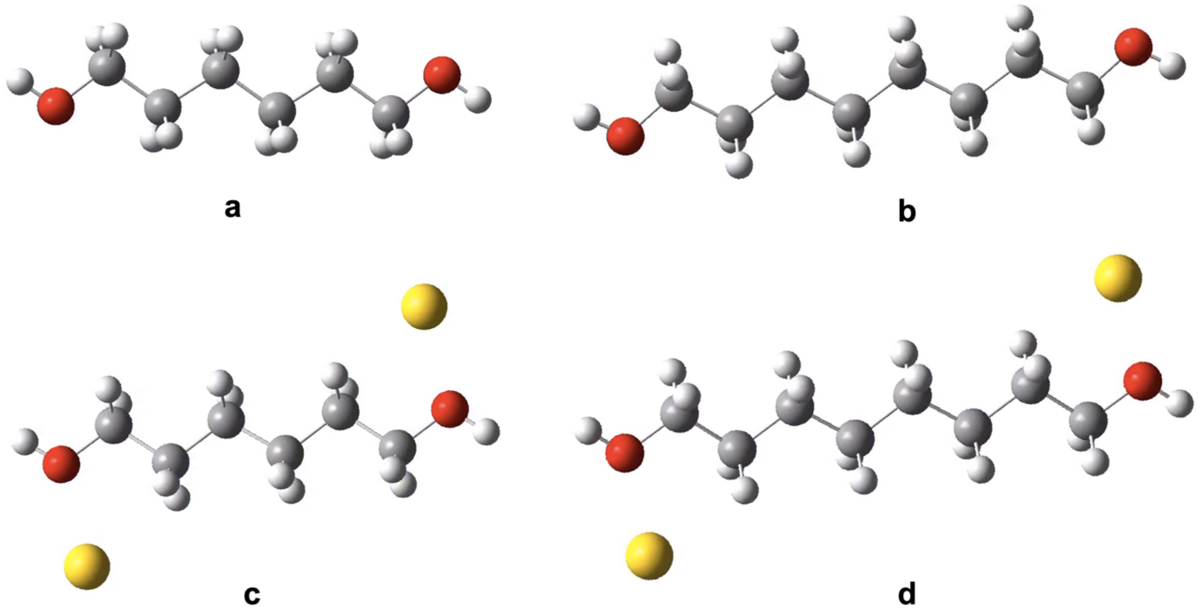

Fig. S1 | Optimized geometries by use of DFT calculations at B3LYP-D3 level of theory. a, 1,6-hexanediol; **b**, 1,8-octanediol; **c**, Au-hexanediol-Au; and **d**, Au-octanediol-Au clusters. O atoms are in red; C atoms are in grey; H atoms are in silver; and gold atoms are in yellow. Both diol molecules adopt a chain structure, and the Au atoms are preferentially bound to the O atoms. Notably, the lowest energy state of Au-diol-Au complexes is a triplet state.

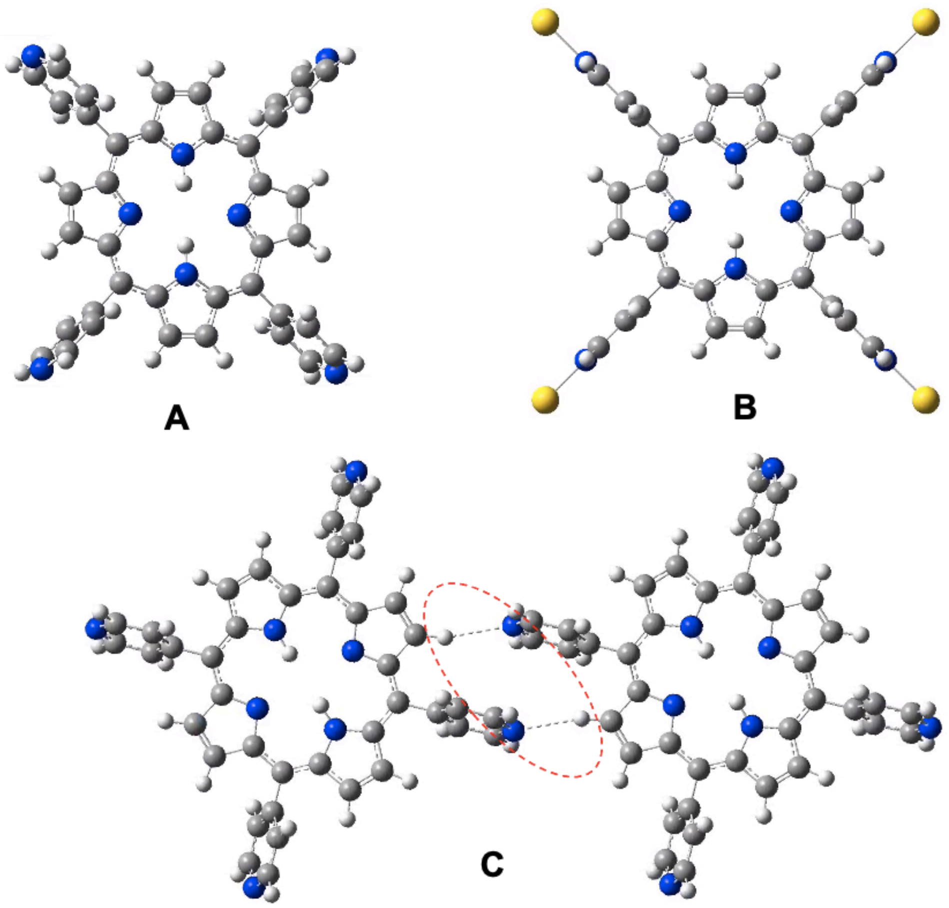

Fig. S2 | Optimized geometries of A, H2TPyP; B, H2TPyP dimer; and C, H2TPyP-Au$_4$ cluster obtained by DFT calculations at B3LYP-D3 level of theory. N atoms are in blue, C atoms are in grey, H atoms are in silver and gold atoms are in yellow. The dashed line highlights the double hydrogen bonds formed between the pyrrole H atoms and the pyridyl N atoms in the H2TPyP dimer. The exposed pyridyl N atoms are expected to be the preferential sites for the growth of Au nanoparticle assemblies.
