# Supplementary material for: Precision engineering of nano-assemblies in superfluid helium by the use of van der Waals forces
Source: Commun Chem. 2024 Jun 4;7:125. doi: 10.1038/s42004-024-01203-5 (PMC11150505; doi:10.1038/s42004-024-01203-5)
Supplement: Supplementary file 3 — Description of Additional Supplementary Files [file 42004_2024_1203_MOESM3_ESM.pdf]

Description of Additional Supplementary Files

File name- Supplementary Data 1

File description- XYZ coordinates of the calculated molecular clusters. Optimized geometries of the molecular clusters. (a) Au2 hexanediol-Au; (b) Au-octanediol-Au; (c) TPyP-Au4; (d) TPyP dimer.
