# Supplementary material for: Precision engineering of nano-assemblies in superfluid helium by the use of van der Waals forces
Source: Commun Chem. 2024 Jun 4;7:125. doi: 10.1038/s42004-024-01203-5 (PMC11150505; doi:10.1038/s42004-024-01203-5)
Supplement: Supplementary file 4 — Supplementary Data 1 [file 42004_2024_1203_MOESM4_ESM.docx]

**Supplementary Data 1.** **Optimized geometries of the molecular clusters.** (a) Au-hexanediol-Au; (b) Au-octanediol-Au; (c) TPyP-Au4; (d) TPyP dimer.

(a). Au-hexanediol-Au

C -2.60732000 -1.84682800 -0.30768200

C -1.80157100 -0.72485000 0.32188100

H -2.74096300 -1.66713400 -1.38139100

H -2.09498500 -2.80830500 -0.17733000

C -0.41418400 -0.57239600 -0.30773900

H -2.36656500 0.20863100 0.21345300

H -1.71277900 -0.91659600 1.39639100

H 0.14515700 -1.51065500 -0.20490800

H -0.51737900 -0.39180700 -1.38496300

C 0.39676100 0.56734500 0.31716300

C 1.78110100 0.72676900 -0.31743600

H 0.50425300 0.38400100 1.39353800

H -0.16580400 1.50419400 0.21917700

C 2.58602200 1.84779500 0.31477100

H 2.34885700 -0.20598100 -0.21768800

H 1.68589100 0.92549100 -1.39013700

H 2.06252400 2.80580300 0.20202800

H 2.73219000 1.65645800 1.38497900

O 3.85995900 1.91515700 -0.35551200

H 4.40137700 2.59827600 0.05490000

O -3.89119300 -1.89683100 0.34742000

H -4.42401000 -2.59778400 -0.04413600

Au -5.34653700 0.28298700 -0.01622700

Au 5.35599600 -0.28448300 0.01464600

(b) Au-octanediol-Au

C 3.87335900 2.12609200 -0.00524300

C 3.09714500 0.81923100 0.07106000

H 3.60451400 2.66256300 -0.91661800

H 3.62835600 2.75461500 0.86040100

C 1.59811500 1.11825800 0.03395500

H 3.36445100 0.17872700 -0.76985600

H 3.34803600 0.29871000 0.99445100

H 1.33119400 1.72875000 0.90706400

H 1.35243300 1.72769500 -0.84211900

C 0.74111100 -0.15096300 0.02404100

C -0.75713800 0.15837100 0.03635600

H 0.99560600 -0.77506700 0.88669100

H 0.98738000 -0.74712700 -0.86090200

C -1.61019600 -1.10877800 0.00404200

H -1.00809800 0.74619200 0.92427000

H -1.00837200 0.78892000 -0.82200400

H -1.34648900 -1.69608000 -0.88193100

H -1.35793000 -1.73657900 0.86537100

C -3.11198900 -0.81541000 -0.00848400

C -3.88333100 -2.12713200 0.02003300

H -3.37619300 -0.25746600 -0.90963500

H -3.37180400 -0.20950400 0.85740900

O -5.29157400 -1.83729800 0.00072900

H -3.61891500 -2.73490500 -0.84943200

H -3.63748500 -2.68442200 0.93191600

H -5.81406300 -2.64268300 0.01822000

O 5.28146000 1.84233000 -0.01378800

H 5.80276100 2.64946900 -0.04878100

Au 5.80814700 -0.42053100 -0.00878700

Au -5.80152600 0.41787200 -0.00760000

(c) TPyP-Au4

C 0.68418700 4.24699500 -0.00003800

C -0.68418200 4.24699600 -0.00001200

C -1.12815800 2.88362200 -0.00002500

C -2.45030200 2.42949500 -0.00003400

C -2.86025700 1.08578100 -0.00003800

C -4.26212100 0.67643900 0.00017500

C -4.26212200 -0.67643300 0.00008000

C -2.86025800 -1.08577700 -0.00000900

N -2.03525700 0.00000100 -0.00004900

C -2.45030500 -2.42949200 0.00000500

C -1.12816200 -2.88362000 0.00002000

C -0.68418700 -4.24699500 0.00002200

C 0.68418200 -4.24699600 0.00002700

C 1.12815800 -2.88362200 0.00002400

C 2.45030200 -2.42949500 0.00003300

C 3.51266800 -3.48415400 0.00001500

C 4.02491200 -3.99172600 1.19578100

C 5.01036200 -4.97079200 1.15250800

C 5.00861500 -4.97251600 -1.15252200

C 4.02309300 -3.99352200 -1.19576500

C 2.86025700 -1.08578100 0.00003400

C 4.26212100 -0.67643900 -0.00013000

C 4.26212200 0.67643300 -0.00012500

C 2.86025800 1.08577700 0.00001400

N 2.03525700 -0.00000100 0.00005100

C 2.45030500 2.42949200 -0.00000400

C 1.12816200 2.88362000 -0.00002000

N 0.00000100 2.10072600 -0.00002400

C 3.51267200 3.48414900 -0.00002900

C 4.02492100 3.99172400 1.19573400

C 5.01037200 4.97078900 1.15245500

C 5.00861800 4.97250600 -1.15257500

C 4.02309400 3.99351300 -1.19581200

C -3.51267300 -3.48414900 0.00001200

C -4.02310300 -3.99352400 1.19578700

C -5.00862600 -4.97251700 1.15253300

C -5.01036300 -4.97077800 -1.15249700

C -4.02491300 -3.99171300 -1.19575900

C -3.51266800 3.48415400 0.00000200

C -4.02492000 3.99173800 -1.19575500

C -5.01036900 4.97080400 -1.15246600

C -5.00860800 4.97250400 1.15256400

C -4.02308500 3.99351000 1.19579100

H 1.33770700 5.10427400 -0.00003900

H -1.33770100 5.10427600 -0.00001300

H -5.11459300 1.33766200 0.00033000

H -5.11459500 -1.33765600 0.00016700

H -1.33770800 -5.10427400 0.00001100

H 1.33770100 -5.10427600 0.00004000

H 3.66239500 -3.62771600 2.14932400

H 5.43358400 -5.38940300 2.05813800

H 5.43048200 -5.39246400 -2.05816600

H 3.65913500 -3.63093400 -2.14930100

H 5.11459300 -1.33766200 -0.00025200

H 5.11459400 1.33765600 -0.00024500

H 0.00000100 1.08763100 -0.00002400

H 3.66240700 3.62771800 2.14927900

H 5.43359700 5.38940200 2.05808200

H 5.43048300 5.39245100 -2.05822100

H 3.65913300 3.63092300 -2.14934600

H -3.65914800 -3.63094300 2.14932600

H -5.43049800 -5.39247000 2.05817200

H -5.43358200 -5.38938300 -2.05813100

H -3.66239100 -3.62769800 -2.14929800

H -3.66240900 3.62773900 -2.14930400

H -5.43359700 5.38942400 -2.05808900

H -5.43046900 5.39244200 2.05821500

H -3.65912100 3.63091200 2.14932100

N -0.00000100 -2.10072600 0.00002200

H -0.00000100 -1.08763100 0.00002000

N 5.49363700 5.45328500 -0.00007000

N 5.49363000 -5.45329200 -0.00001400

N -5.49363700 -5.45328600 0.00002000

N -5.49363000 5.45329300 0.00006400

Au -7.12625900 7.09528200 0.00009700

Au 7.12626900 7.09527200 -0.00008600

Au 7.12625900 -7.09528200 -0.00002000

Au -7.12626900 -7.09527200 0.00000900

(d) TPyP dimer

C -1.82319000 0.92061400 -0.01894500

C -0.45584900 0.91311800 0.02278800

C -0.01749700 -0.45160000 0.03030200

C 1.30512100 -0.91115200 0.06528800

C 1.70485100 -2.25985700 0.08254900

C 3.10317800 -2.68038200 0.04227500

C 3.10083200 -4.03304500 0.04661500

C 1.69559600 -4.43273900 0.08904600

N 0.87383000 -3.34212200 0.10343200

C 1.28003100 -5.77613300 0.07855900

C -0.04753600 -6.22199100 0.04085100

C -0.50034900 -7.58177900 0.03380800

C -1.86773000 -7.57486500 -0.01352600

C -2.30658000 -6.21058700 -0.02212000

C -3.62906000 -5.75206700 -0.06075200

C -4.69585400 -6.79910400 -0.08170500

C -5.46848300 -7.02338700 -1.22427200

C -6.45025400 -8.01166900 -1.19587800

C -5.96840400 -8.55314300 0.96451900

C -4.96171200 -7.59240400 1.03753300

C -4.02957900 -4.40323400 -0.07605600

C -5.42758300 -3.98498200 -0.03686600

C -5.42057000 -2.63223400 -0.03591500

C -4.01829400 -2.22844300 -0.07486700

N -3.19984300 -3.32000200 -0.09239000

C -3.60376200 -0.88362900 -0.05954800

C -2.27662700 -0.43915300 -0.02437200

N -1.15105400 -1.22762000 0.00373800

C -4.65958200 0.17451700 -0.07737200

C -5.43228500 0.40817600 -1.21800100

C -6.40358700 1.40664800 -1.18634000

C -5.91132800 1.94063700 0.97357300

C -4.91456700 0.96935800 1.04329300

C 2.33244300 -6.83783700 0.10857400

C 2.60196800 -7.62905100 -1.01201000

C 3.58498400 -8.61054100 -0.92759500

C 4.03761800 -8.09431300 1.24694600

C 3.07838800 -7.08496300 1.26237200

C 2.37255300 0.13535900 0.08031200

C 3.15047100 0.36059300 1.21904400

C 4.13304200 1.34777100 1.18492200

C 3.64215300 1.88679200 -0.97392700

C 2.63413200 0.92705000 -1.04104600

H -2.46978900 1.78199100 -0.04482300

H 0.20025400 1.76730400 0.04729800

H 3.95960100 -2.02678200 -0.00570800

H 3.96113900 -4.68622100 -0.00175300

H 0.14608200 -8.44321500 0.06166200

H -2.52326900 -8.42940600 -0.04090800

H -5.30399500 -6.43772800 -2.12092100

H -7.05748400 -8.20187300 -2.07639200

H -6.19207400 -9.17466300 1.82703200

H -4.39759000 -7.45681800 1.95274000

H -6.28323100 -4.63989000 0.00462800

H -6.26938800 -1.96856000 0.00647600

H -1.15555300 -2.24080300 0.00451800

H -5.27579000 -0.17802900 -2.11572200

H -7.01065000 1.60425700 -2.06533600

H -6.12648800 2.56355600 1.83723900

H -4.34976500 0.82697800 1.95704700

H 2.05662100 -7.47561600 -1.93566300

H 3.81549900 -9.22959100 -1.79027700

H 4.62927900 -8.30019600 2.13403700

H 2.91412500 -6.49712100 2.15730300

H 2.98966400 -0.22413500 2.11694400

H 4.74431600 1.53865100 2.06251600

H 3.86254300 2.50686900 -1.83833600

H 2.06588800 0.79080100 -1.95360800

N -1.17270300 -5.43405200 0.00856700

H -1.16603900 -4.42084600 0.00815800

N -6.65131000 2.16857200 -0.11611100

N -6.70827000 -8.77216300 -0.12707200

N 4.29722700 -8.85215100 0.17795400

N 4.38722800 2.10658300 0.11396600

C 10.82748200 -7.03755100 -0.03356800

C 12.19486000 -7.04447900 0.01384000

C 12.63370000 -8.40876000 0.02236300

C 13.95617400 -8.86729200 0.06102700

C 14.35668500 -10.21612900 0.07625000

C 15.75468800 -10.63438500 0.03709700

C 15.74766800 -11.98713400 0.03604000

C 14.34538800 -12.39091900 0.07489000

N 13.52694200 -11.29935700 0.09245700

C 13.93084900 -13.73573000 0.05944600

C 12.60371200 -14.18019500 0.02417100

C 12.15026700 -15.53995800 0.01861800

C 10.78292800 -15.53245000 -0.02317700

C 10.34458600 -14.16772800 -0.03060900

C 9.02197200 -13.70816500 -0.06561700

C 7.95453400 -14.75466900 -0.08075500

C 7.17666200 -14.97982200 -1.21953500

C 6.19408400 -15.96699700 -1.18551900

C 6.68488100 -16.50616600 0.97331300

C 7.69290500 -15.54643500 1.04053900

C 8.62225000 -12.35945600 -0.08279800

C 7.22392500 -11.93892700 -0.04253200

C 7.22627900 -10.58626400 -0.04675900

C 8.63151800 -10.18657400 -0.08914600

N 9.45327800 -11.27719400 -0.10357500

C 9.04709000 -8.84318200 -0.07853900

C 10.37465800 -8.39733600 -0.04072400

N 11.49981800 -9.18528500 -0.00843400

C 7.99468500 -7.78147100 -0.10854500

C 7.24888200 -7.53419500 -1.26240200

C 6.28965500 -6.52484300 -1.24696800

C 6.74201600 -6.00889800 0.92769700

C 7.72502300 -6.99039900 1.01210700

C 14.98666100 -14.79388300 0.07724100

C 15.24169700 -15.58864000 -1.04347300

C 16.23844900 -16.55993000 -0.97377800

C 16.73060400 -16.02611000 1.18620000

C 15.75930700 -15.02763500 1.21789000

C 15.02297400 -7.82026400 0.08210800

C 15.79555200 -7.59607100 1.22472800

C 16.77732900 -6.60779200 1.19645300

C 16.29558300 -6.06615400 -0.96392500

C 15.28888800 -7.02688100 -1.03705800

H 10.18106000 -6.17610800 -0.06140200

H 12.85040500 -6.18994500 0.04131300

H 16.61034200 -9.97948000 -0.00430400

H 16.59648400 -12.65080900 -0.00636000

H 12.79685900 -16.40134200 0.04446100

H 10.12682000 -16.38663000 -0.04778200

H 7.33750900 -14.39503400 -2.11738900

H 5.58284400 -16.15781400 -2.06315200

H 6.46445200 -17.12630000 1.83767100

H 8.26111200 -15.41025100 1.95313300

H 6.36749800 -12.59252500 0.00538800

H 6.36597800 -9.93308400 0.00166600

H 11.49314700 -10.19849100 -0.00809200

H 7.41325400 -8.12192300 -2.15738800

H 5.69810600 -6.31884300 -2.13410600

H 6.51139700 -5.38995800 1.79043000

H 8.27025600 -7.14395200 1.93580900

H 14.67694100 -15.44618700 -1.95724300

H 16.45364800 -17.18278400 -1.83748100

H 17.33762300 -16.22379000 2.06521100

H 15.60277100 -14.44149800 2.11564800

H 15.63101800 -8.18179600 2.12132600

H 17.38452000 -6.41765800 2.07701100

H 16.51929600 -5.44457100 -1.82638100

H 14.72480800 -7.16239500 -1.95230100

N 11.47814600 -13.39171800 -0.00393400

H 11.48265200 -12.37853500 -0.00463500

N 6.02991200 -5.76714400 -0.17790900

N 5.93985000 -16.72587900 -0.11462600

N 16.97837500 -16.78795300 0.11592400

N 17.03539900 -5.84722000 0.12771600
